# Epidemiology of Marek’s Disease Virus Type 2 and the Molecular and Pathology Characterization of Virus Isolates in China (2015–2024)

**DOI:** 10.1155/tbed/5910446

**Published:** 2026-04-10

**Authors:** Yumeng Lin, Mingxue Hu, Chuyan Wu, Hangqiong Lu, Mengyun Chen, Changjun Liu, Hongyu Cui, Yongzhen Liu, Xiaole Qi, Suyan Wang, Yuntong Chen, Yulu Duan, Yulong Gao, Yanping Zhang

**Affiliations:** ^1^ Avian Immunosuppressive Diseases Division, State Key Laboratory of Animal Disease Control and Prevention, Harbin Veterinary Research Institute, The Chinese Academy of Agricultural Sciences, Harbin, 150069, China, caas.cn; ^2^ Jiangsu Co-innovation Center for Prevention and Control of Important Animal Infectious Disease and Zoonoses, Yangzhou, 225009, China

**Keywords:** epidemiological survey, genetic evolutionary analysis, Marek’s disease virus, pathogenicity, serotype 2

## Abstract

Marek’s disease (MD) continues to cause significant economic losses in the global poultry industry. During routine MD surveillance in China, MD virus type 2 (MDV‐2), which has the potential for widespread dissemination, was detected. In this study, 2842 suspected MD samples were collected from 237 poultry flocks vaccinated against MD across 21 provinces and four municipalities in China. Among these, 534 samples (18.79%) from 95/150 flocks (63.29%) tested positive for MDV‐2, with detections reported in all provinces and municipalities. A total of 54 MDV‐2 strains were isolated and further characterized. The genomes of four isolates, SW20, JLWK1501, LB3, and ZH4, were sequenced and analyzed. Comparative analysis with reference MDV‐2 strains (SB‐1, 301B/1, and HPRS24) showed that 33 genes in the Chinese isolates differed in length due to insertions or deletions, whereas 27 genes exhibited single‐nucleotide polymorphisms (SNPs) without length variation. Homology analysis demonstrated that the four Chinese isolates were more closely related to the US strain 301B/1 than to the US strain SB‐1 or the UK strain HPRS24. Point mutations resulted in SNP differences in 18 genes between strain 301B/1 and the Chinese isolates. Additionally, variations in the lengths of four genes (*ORF3*, *ORF413*, *R-LORF1*, and *R-LORF9*) were observed among the four Chinese isolates, indicating marked variability in these genomic regions. These findings align with alterations reported in circulating MDV‐2 strains in China. Pathogenicity assessment of strain SW20 in specific‐pathogen‐free chickens showed that the strain induced inflammatory liver nodules, thymic atrophy, and splenic enlargement. This study highlights the expanding distribution of MDV‐2 in China and confirms its ability to induce inflammatory responses, representing the first systematic epidemiological investigation of MDV‐2 in the country. The findings provide an important reference for MD control efforts and emphasize the ongoing need for active surveillance.

## 1. Introduction

Marek’s disease (MD) is a contagious lymphoproliferative condition in poultry, characterized by T‐cell lymphoma and immunosuppression caused by MD virus (MDV) [1]. This virus poses a substantial threat to commercial chicken production systems [2]. MDV is categorized into three serotypes—MDV type 1 (MDV‐1), MDV type 2 (MDV‐2), and herpesvirus of turkeys (HVT)—based on serological properties [3]. Among these, only MDV‐1 is oncogenic [4], inducing lymphomas and neurological manifestations such as paralysis and blindness due to neoplastic cell infiltration [5, 6], whereas MDV‐2 and HVT are nonpathogenic and widely utilized as vaccines [7, 8]. Despite extensive vaccination, increasing reports of vaccine failure have emerged in several countries, including Ethiopia, Iran, Turkey, Thailand, and China [9–11, 12–14], contributing to economic losses exceeding $1 billion annually [15, 16]. Growing evidence suggests that environmental pressures, expanding poultry populations, imperfect vaccine‐induced immunity [17], and intensified production systems [18, 19] have accelerated the emergence of increasingly virulent or immune‐evasive MDV‐1 strains [20, 21]. These factors have enabled persistent viral transmission in vaccinated flocks and increased the transboundary risk associated with MD.

Given the pathogenic nature of MDV‐1, research has largely focused on this strain. In 1972, MDV‐2 HN‐1 was first detected in healthy flocks [22]. Since then, MDV‐2 strains such as SB‐1, 301B/1, HPRS24, Z4, GM‐1, and 471B/1 have been reported in several countries [23–26]. These strains grow well in chicken embryo fibroblasts (CEFs) and form plaques that differ from those formed by MDV‐1 and HVT [27]. The lack of viral internal antigen expression, cytolysis, and lymphomagenesis in MDV‐2 explains why MDV‐2 strains are considered nononcogenic [26, 27]. Notably, strains 301B/1 and SB‐1 provide protection against MD [28–30], with SB‐1 used as a vaccine through the 1980s and early 1990s and becoming common in commercial flocks [31–33]. Since the early 21st century, MDV‐2 has been replaced by vaccines such as CVI988, CVI988/HVT, and 814 due to rising MDV virulence; however, immunization failures persist [34–36]. The emergence of highly virulent MDV‐1 strains that overcome vaccine protection has caused outbreaks [37]. During ongoing MD surveillance in China, MDV‐2 was detected in suspected MD cases, whereas MDV‐1 was not [38]. Despite this, the role of MDV‐2 in MD pathogenesis remains unclear.

To evaluate the epizootic potential of MDV‐2 and track its genetic evolution, a systematic epidemiological investigation has been conducted in chickens with suspected MD in China since 2015. MDV‐2 is currently prevalent in China, and the detected strains are closely related to 301B/1. To our knowledge, few studies have focused solely on the pathogenicity of MDV‐2. Therefore, we examined the epidemiological characteristics, genetic evolution, and pathogenicity of MDV‐2 in China. The four detected MDV‐2 strains from different provinces and municipalities in this study were compared with the complete genome sequences of MDV‐2 reference strains SB‐1, 301B/1, and HPRS24. Our findings highlight its prevalence and impact, providing an essential basis for prevention and control.

## 2. Materials and Methods

### 2.1. Cells, Viruses, Antibodies

CEFs were prepared from 10‐day‐old SPF chicken embryos [39]. The SB‐1 strain was stored in our laboratory, and the CVI988 virus was purchased commercially.

Representative MDV‐1 and MDV‐2 strains, CVI988 (Number DQ530348) and SB‐1 (Number HO840738), were used as positive controls for the indirect immunofluorescence assay (IFA) to identify isolates. Monoclonal antibodies specific to MDV gE, MDV‐1gI, and HVT gB were prepared in our laboratory.

### 2.2. Clinical Samples and Viral Identification

From 2015 to 2024, 2842 samples were collected from 237 flocks in 21 provinces and four municipalities across China, including Heilongjiang, Hebei, and Hunan. Samples included feather pulp, liver tissue, and peripheral blood leukocytes (PBLs) from chickens vaccinated with 814, CVI988, or CVI988/HVT and suspected of having MD.

DNA was extracted using the method described by Zhang et al. [14]. The *meq* gene of MDV‐1 and *ORF873* gene of MDV‐2 were amplified by polymerase chain reaction (PCR) using primers listed in Table S1 [38]. Positive samples were stored in our laboratory at −80°C.

### 2.3. Virus Isolation, Identification, DNA Purification, Sequencing, and Analysis

PBLs were isolated from anticoagulated blood samples from suspected MD cases using the Ficoll‐Paque method [40]. Virus isolation was performed by inoculating PBLs into M199 supplemented medium with 5% fetal bovine serum. Cultures were blindly passaged for two to three rounds until cytopathogenic effects (CPEs) appeared. The *meq* gene of MDV‐1, *ORF873* gene of MDV‐2, *US3* gene of HVT, *VP3* gene of chicken infectious anemia virus (CIAV), *env* gene of avian leukosis virus (ALV‐A, ALV‐B, and ALV‐J), and *LTR* gene of reticuloendotheliosis virus (REV) were amplified by PCR using the primers listed in Table S1 [38]. Amplified products were mixed with cell‐freezing medium at the appropriate ratio and stored in liquid nitrogen.

To distinguish field isolates from vaccine strains, whole‐genome sequencing (WGS) was performed on MDV‐2 isolates. WGS selection criteria included clear CPEs, sufficient viral DNA (>100 ng/µL), and diverse geographic origins. Viral DNA collected from poultry farms was isolated from infected CEFs using a micrococcal nuclease procedure to degrade cellular DNA. Proteinase K was then used to digest the viral capsid and release viral DNA [41]. Purified DNA was sequenced using the Illumina HiSeq2000 platform (San Diego, CA, USA). After removing host sequences, assemblies were optimized based on paired‐end and overlap relationships by mapping reads to contigs. Sequences were validated using Sanger sequencing to confirm repetitive or ambiguous regions, combined with NCBI BLAST and Geneious (Auckland, New Zealand).

The assembled sequences were compared with MDV‐1 reference strains CVI988 (Number DQ530348), GA (Number AF147806), Md5 (Number AF243438) and MDV‐2 reference strains SB‐1 (Number HO840738), HPRS24 (Number AB049735), 301B/1 (Number MH939248), and HVT (Number AF291866). These sequences were aligned, and phylogenomic relationships were analyzed using MAFFT software [42]. A phylogenetic tree was constructed using the neighbor‐joining method with 1000 bootstrap replicates and visualized using MEGA version 10 software [43].

### 2.4. Pathogenicity

Animal experiments were conducted to analyze the pathogenicity of MDV‐2. Fifty‐four 1‐day‐old SPF whiteleghorn chickens were obtained from the Harbin Veterinary Research Institute (Harbin, China). The chickens were randomly assigned to three groups, housed in separate isolators under negative pressure, and provided adequate water, food, and a comfortable environment. On Day 1, chickens in different groups were intraperitoneally injected with either 8000 PFU of SW20 or SB‐1 per chicken. The remaining chickens were injected with the same diluent as a negative control.

To assess the effect of MDV‐2 infection, clinical signs and mortality were monitored daily until 90 days postchallenge (dpc). At 7, 14, 21, and 28 dpc, three chickens per group were humanely euthanized, and at 90 dpc, six chickens per group were humanely euthanized for the collection of the thymus, spleen, bursa of Fabricius, and feather pulps. Immune‐organ and body weights were recorded. At the end of the study, all animals were euthanized for necropsy, and the same samples were collected on the same days as described previously. Tissues of liver and testes were fixed in 10% neutral‐buffered formalin, processed, and embedded in paraffin wax for histopathology [44].

Viral load in homogenized feather pulp and immune organs was quantified using quantitative PCR (qPCR). qPCR was performed using a fluorescent qPCR instrument (QuantStudio 5, Biosystems, USA) following previously described methods [45, 46].

### 2.5. Statistical Analyses

Data on body weight, immune organ index, and viral load among the groups at each time point were analyzed using two‐way ANOVA. The analysis was performed with GraphPad Prism (Version 8.0.1) to assess differences among the groups. Statistical significance was set at *p*  < 0.05.

### 2.6. Ethics Statement

In accordance with the recommendations published by the Ministry of Science and Technology of China regarding the Guide for the Care and Use of Laboratory Animals, this study was approved by the Animal Ethics Committee of the Harbin Veterinary Research Institute, Chinese Academy of Agricultural Sciences. The study was conducted in compliance with animal ethics guidelines (Approval Number: SYXK [Hei] 230815001‐GR). All procedures were performed under approved protocols, and appropriate measures were taken to minimize discomfort and suffering of the animals.

## 3. Results

### 3.1. Nationwide Prevalence of MDV‐2

From 2015 to 2024, 40.92% (1163/2842) and 18.79% (534/2842) of the collected samples were positive for MDV‐1 and MDV‐2, respectively. Additionally, 72.85% (389/534) of the MDV‐2‐positive samples were coinfected with MDV‐1 (Figure 1A). The 534 MDV‐2‐positive samples were obtained from 95 of 150 chicken flocks (63.29%) across the 21 provinces and four municipalities in China (Figure 1B, C).

Figure 1Epidemiological surveys. (A) Detection results for 2842 clinical samples collected from 150 MD‐vaccinated chicken flocks across 21 provinces and 4 municipalities in China. (B) Geographic distribution of MDV‐2‐positive samples collected in China from 2015 to 2024. Different shades of red indicate the percentage of MDV‐2‐positive flocks, whereas gray areas represent provinces where no samples were detected in this study. (C) Bar plot illustrating the number of MDV‐2–positive flocks collected from 21 provinces and 4 municipalities in China from 2015 to 2024. Negative flocks are shown in red, and positive flocks are shown in blue.

**Figure:**
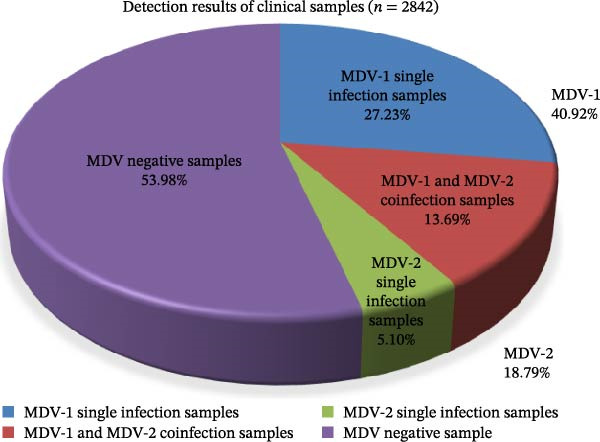
(A)

**Figure:**
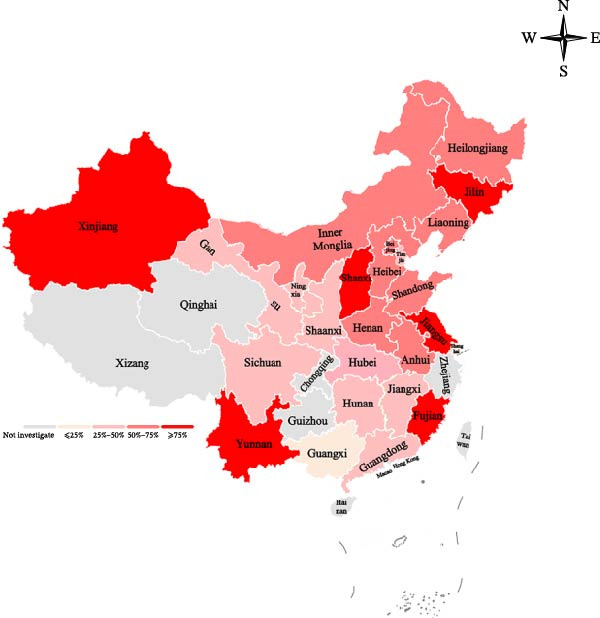
(B)

**Figure:**
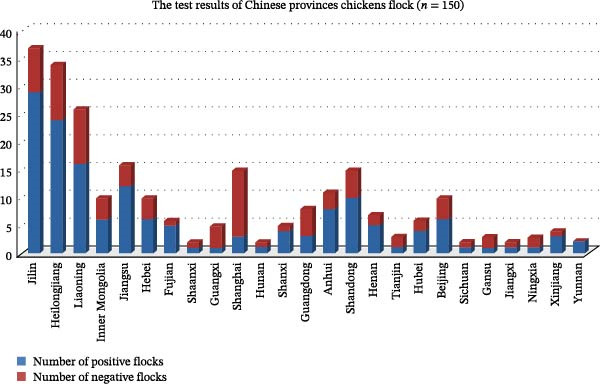
(C)

Sixty‐six CEF cultures inoculated with PBMCs from MDV‐2‐infected chickens exhibited typical MD CPEs after 5 days. All 66 isolates were positive for MDV‐2, with 54 isolates belonging to MDV‐2 alone, 9 coinfected with MDV‐2 and HVT, and 3 coinfected with MDV‐1 and MDV‐2, as confirmed by PCR. Detailed information is provided in Table S2. In addition, comprehensive screening for other avian pathogens, including CIAV, ALV‐A, ALV‐B, ALV‐J, REV, and FAdV, yielded negative results.

**Figure 2:**
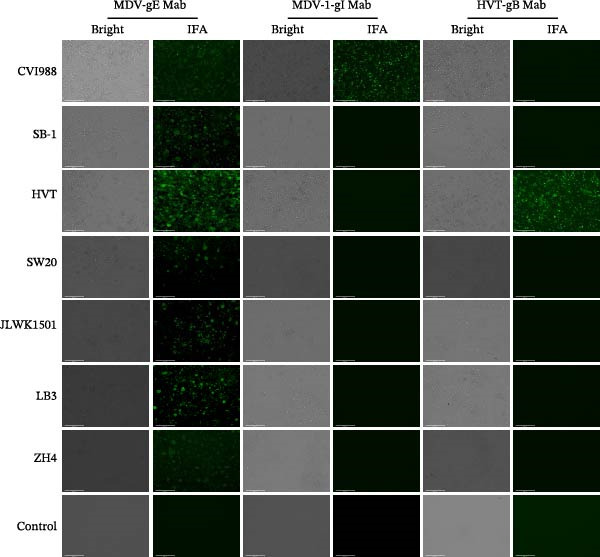
Detection of antigen in infected cells of four strains (SW20, JLWK1501, LB3, and ZH4) using indirect immunofluorescence assay (IFA). CVI988, SB‐1, and HVT served as positive controls for MDV Mab (gE), MDV‐1 Mab (gI), and HVT‐specific Mab (gB), respectively. Cells were fixed and incubated with monoclonal antibodies specific to Marek’s disease virus (MDV), followed by a fluorescein isothiocyanate (FITC)‐conjugated secondary antibody. Fluorescence microscopy showed green fluorescence in the four strains with MDV Mab (gE), but no green fluorescence with MDV‐1 Mab (gI) or HVT‐specific Mab, confirming that the four strains belong to MDV‐2. Scale bar = 125 μm.

Four isolates (SW20, JLWK1501, LB3, and ZH4) were selected from distinct provinces in China and further evaluated by IFA; all tested positive for MDV‐2 (Figure 2). Overall, MDV‐2 strains were identified in chicken flocks across all 21 provinces and four municipalities, confirming the widespread distribution of MDV‐2 in China.

### 3.2. Genome Structure of MDV‐2 and Phylogenomic Relatedness

To further explore the detected strains prevailing in China and differentiate field isolates from vaccine strains, the genomes of four strains (SW20, JLWK1501, LB3, and ZH4) obtained from multiple provinces and municipalities were sequenced. The assembled genomes of these strains were ~164,666–164,918 base pairs in length and contained six subgenomic regions (TRL‐unique long (UL)‐IRL‐IRS‐US‐TRS). The GenBank accession numbers and genome regions are presented in Table 1.

**Table 1:** Comparison of subgenomic regions in the genome of strains SW20, JLWK1501, LB3, and ZH4.

Subgenomic region	SW20 No. PX425604			JLWK1501 No. PX425602			LB3 No. PX425603			ZH4 No. PX425605		
	Start	End	Length (bp)	Start	End	Length (bp)	Start	End	Length (bp)	Start	End	Length (bp)
a‐like^A^	1	217	216	1	219	218	1	218	217	1	218	217
TRL	218	12,067	11,849	220	12,037	11,817	219	12,067	11,848	219	12,068	11,849
UL	12,068	121,813	109,745	12,038	121,787	109,749	12,068	121,814	109,746	12,069	121,817	109,748
IRL	121,814	133,663	11,849	121,788	133,605	11,817	121,815	133,663	11,848	121,818	133,728	11,910
a‐like^A^	133,664	134,074	410	133,606	133,824	218	133,664	133,857	193	133,729	133,958	229
IRS	134,075	143,329	9254	133,825	143,081	9256	133,858	143,100	9242	133,959	143,199	9240
US	143,330	155,440	12,110	143,082	155,203	12,121	143,101	155,218	12,117	143,200	155,316	12,116
TRS	155,441	164,695	9254	155,204	164,460	9256	155,219	164,461	9242	155,317	164,557	9240
a‐like^A^	164,696	164,918	222	164,461	164,666	205	164,462	164,721	259	164,558	164,769	211

^A^The discrepancies in the a‐like sequences of HPRS24 are most likely due to the repetitive nature of these sequences and the sequencing method (Sanger) used.

Reference strains of MDV‐1 (CVI988, GA, and Md5), MDV‐2 (SB‐1, HPRS24, and 301B/1), and HVT, along with the four Chinese strains (SW20, JLWK1501, LB3, and ZH4), were aligned and analyzed. Phylogenetic analysis showed that the Chinese strains were more closely related to the US MDV‐2 strain 301B/1 (Figure 3A). The homology between the four Chinese strains was high, with 99.5%–99.8% similarity to 301B/1. However, similarity to HPRS24, the MDV‐2 strain isolated in the United Kingdom, was lower, ranging from 97.5% to 97.7%. The primary differences between the Chinese strains and the MDV‐2 reference strains occurred in the repeat long, repeat short, and unique long (UL) regions (Figure 3B). In comparison with the 301B/1 genome, size differences in 12 genes (*ORF3*, *ORF413*, *R-LORF1*, *R-LORF9*, *R-LORF3*, *R-LORF5*, *R-LORF6*, *R-LORF8*, *R-SORF1*, *SORF2*, *pp24*, and *pp38*) were attributed to insertion–deletions (INDELs), resulting in frameshifts or alternative start sites in coding sequences (Table 2).

Figure 3Sequence comparison analysis. (A) Phylogenetic tree showing the evolutionary relationships of SW20, JLWK1501, LB3, and ZH4 with MDV‐2 reference strains (SB‐1, 301B/1, and HPRS24). The dendrogram was generated using the neighbor‐joining method with 100 bootstrap resamplings. (B) Genome‐wide comparison illustrating sequence divergence between Chinese isolates and reference strains. Most variability occurred in the repeat long (RL), repeat short (RS), and unique long (UL) regions. The right‐side color bar indicates percent identity between each genomic region and the 301B/1 reference genome, with color gradients representing decreasing homology. (C) Gene‐specific variation among the four Chinese isolates. Four genes (*ORF3*, *ORF413*, *R-LORF1*, and *R-LORF9*) exhibited distinct length differences, indicating high variability in these loci. Gene‐length variation among four highly variable loci (*ORF3*, *ORF413*, *R-LORF1*, and *R-LORF9*). Insertions and deletions produced strain‐specific differences in coding sequence length. Each strain is represented by a distinct color, and horizontal bars show gene lengths relative to the 301B/1 genome.

**Figure:**
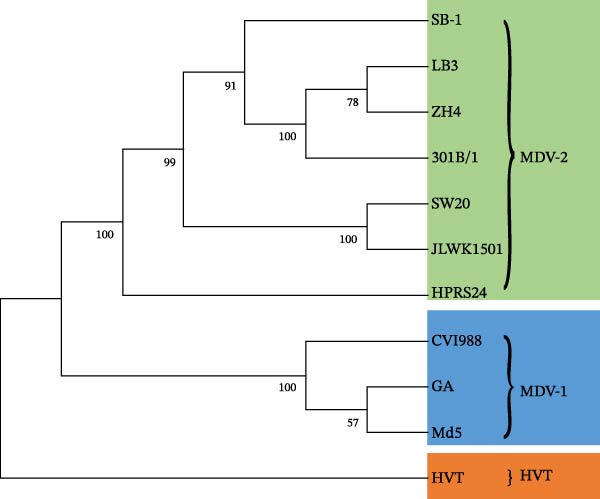
(A)

**Figure:**
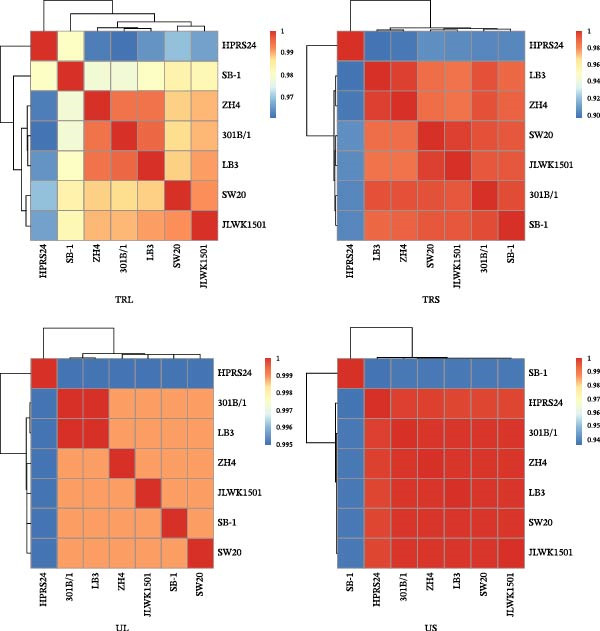
(B)

**Figure:**
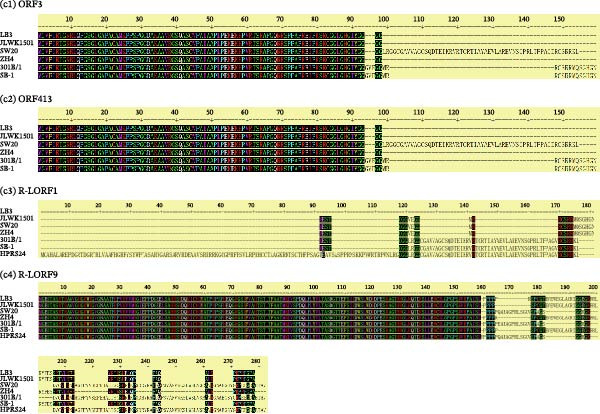
(C)

**Table 2:** Comparison of genes that have different aa lengths among MDV‐2 genomes^A^.

ORFs	Length of coding sequence (aa)^B^						
	301B/1	SB‐1	HPRS24	SW20	JLWK15‐1	LB3	ZH4
R‐LORF1	63	—	176	No	25	25	25
ORF3	112	—	96	151	95	95	95
R‐LORF3	116	—	—	—	108	—	—
R‐LORF5‐1	96	97	102	—	110	101	—
R‐LORF6	130	—	—	—	—	57	—
R‐LORF7	120	131	131	—	—	—	—
R‐LORF9	262	224	263	261	223	225	224
R‐LORF8	327	120	—	132	132	—	—
pp24	175	159	—	165	167	171	—
LORF3	55	—	273	—	—	—	—
LORF2	363	—	437	—	—	—	—
UL3.5	84	—	89	—	—	—	—
UL5	857	—	858	—	—	—	—
UL9	839	—	875	—	—	—	—
UL25	582	—	594	—	—	—	—
UL28	787	—	811	—	—	—	—
UL30	1211	—	1190	—	—	—	—
UL36	3068	—	3064	—	—	—	—
ORF242	217	—	216	—	—	—	—
UL37	1046	—	1040	—	—	—	—
UL47	811	—	810	—	—	—	—
LORF4	268	268	222	—	—	—	—
UL56	124	—	129	—	—	—	—
ORF368	64	—	84	—	—	—	—
pp38	249	—	221	239	241	245	—
R‐LORF5‐2	96	102	102	—	110	101	—
R‐LORF2‐2	181	—	—	—	—	25	—
ORF413	112	—	—	151	95	95	95
US8	518	—	488	—	—	—	—
SORF2	114	—	94	94	—	—	94
SORF5	85	—	83	—	—	—	—
ORF517	100	—	No	—	—	—	—
R‐SORF1	92	91	359	90	125	128	47

*Note:* — Indicates consistency with gene lengths of the reference strain 301B/1 genes. No = Indicates the absence of a particular gene or mutation in the corresponding MDV‐2 strain.

^A^The genes in repeat regions (R‐LORF1, ORF3, RLORF7, R‐LORF9, and R‐LORF8 in the repeat long and R‐SORF1 in the repeat short) are listed only once.

^B^aa = amino acids.

Twenty‐seven genes exhibited 60 single‐nucleotide polymorphisms (SNPs), as detailed in Table S3. A comparison with the 301B/1 genome revealed that SNPs occurred in 18 genes (*ICP4*, *LORF4*, *ORF473*, *R-LORF4*, *UL1*, *UL8*, *UL17*, *UL16*, *UL22*, *UL33*, *UL36*, *UL43*, *UL47*, *UL52*, *UL56*, *US3*, *US7*, and *US10*), causing nonsynonymous amino acid substitutions without affecting gene length (Table 3).

**Table 3:** Analysis of single‐nucleotide polymorphisms of 18 genes among the SW20, JLWK1501, LB3, and ZH4 genomes with the corresponding genes in the 301B/1 genome.

Genes	Amino acid sequence						Position in amino acid	Genes	Amino acid sequence					Position in amino acid
	301B/1	SW20	JLWK1501		Hrb09LB3	Hrb09ZH4			301B/1	SW20	JLWK1501	Hrb09LB3	Hrb09ZH4	
ICP4	L	P		P	—	—	252	UL33	A	—	T	—	—	3
ICP4	H	—		L	—	—	260	UL33	T	—	A	—	—	90
ICP4	T	—		S	—	—	261	UL36	E	V	V	—	—	842
ICP4	C	R		R	—	—	1982	UL43	A	—	T	—	—	184
LORF4	C	R		R	—	—	79	UL47	A	—	—	—	V	611
ORF473	N	S		S	—	—	101	UL52	A	T	—	—	—	139
R‐LORF4	V	—		G	—	—	39	UL52	R	K	K	—	—	349
R‐LORF4	L	V		V	—	—	75	UL52	I	—	M	—	—	753
UL1	H	R		R	—	—	2	UL56	A	V	—	—	—	55
UL8	R	—		Q	—	—	555	US3	P	—	—	—	L	31
UL17	R	K		—	—	—	450	US7	M	I	I	—	—	346
UL16	V	M		—	—	—	349	US10	C	Y	Y	—	—	203
UL22	N	S		S	—	—	762							

*Note:* — Indicates consistency with gene lengths of the reference strain 301B/1 gene.

Notably, the lengths of *ORF3*, *ORF413*, *R-LORF1*, and *R-LORF9* varied among the Chinese strains, indicating marked variability in these regions (Figure 3C).

### 3.3. Pathogenicity

To assess the pathogenicity and replication ability of MDV‐2 in vivo, the SW20 strain was used in animal experiments. None of the chickens in any group died by 90 dpc. At 90 dpc, all six chickens in each group were subjected to autopsy. Compared to chickens in the control group, two of the three male chickens infected with the SW20 strain showed a 2.5‐times enlargement of the testes at 90 dpc (Figure 4A), with a rate of 66.7%. Figure 4A shows unilateral testes from three male chickens per group (SW20, SB‐1, and negative control), with control chickens explicitly included for comparison. At the same sampling time (90 dpc), inflammatory cell infiltration was observed in the interstitial tissue of the testes infected with SW20 (Figure 4B) compared to the control (Figure 4C). Additionally, the liver of one SW20‐infected chicken exhibited edema, congestion, and grayish‑white necrotic foci (Figures 4D–F), at a rate of 16.7% (1/6). No visible lesions were observed in SB‐1‐infected or control chickens. Histopathological examination revealed multiple inflammatory cell infiltrates in the parenchyma, accompanied by perrilobular hepatocellular atrophy, manifested as reduced cell size and cytoplasmic shrinkage in all infected birds. Furthermore, the degree of inflammatory cell infiltration was more severe in the SW20 group, with infiltration also observed around the central vein (Figures 4G–I). To investigate growth retardation and immune organ damage (thymus, spleen, and bursa) caused by MDV‐2 inoculation, the body weights of chickens and ratio of immune organ weight to body weight were analyzed for all groups. Body weights did not differ significantly between groups (Figure 5A). However, compared to the negative control, the SW20 strain induced more severe thymus atrophy than the SB‐1 strain (*p*  < 0.05) at 14 dpc (Figure 5B) and caused spleen enlargement (Figure 5C). The bursa showed no significant damage (Figures 5C, D).

Figure 4Anatomical and histological lesions. (A) Representative gross appearance of testes from male chickens in the SW20, SB‐1, and negative control (NC) groups. Three male chickens were included per group, and one unilateral testis from each chicken is shown. In the SW20 group, two of three male chickens (2/3) exhibited testicular enlargement at 90 dpc. Bilateral testes from the same individual showed consistent gross changes; therefore, only one testis per chicken is displayed. Control chickens are explicitly included for comparison. (B) Inflammatory cell infiltration in the interstitium of testicular tissue from chickens infected with the SW20 strain at 90 dpc. (C) Testicular tissue from the negative control group at 90 dpc. (D) White, solid nodules approximately 1–3 mm in diameter in the liver of a chicken infected with SW20 (indicated by circle). (E) Liver of a chicken in the SB‐1 group showing no visible clinical lesions. (F) Liver tissue from the negative control group. (G) Numerous inflammatory cells concentrated in the liver tissue of a chicken infected with SW20. (H) A small number of inflammatory cell infiltrates in the liver tissue of a chicken infected with SB‐1. (I) Liver tissue from the negative control group.

**Figure:**
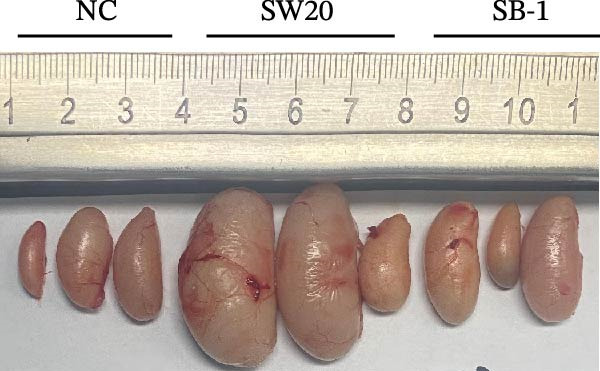
(A)

**Figure:**
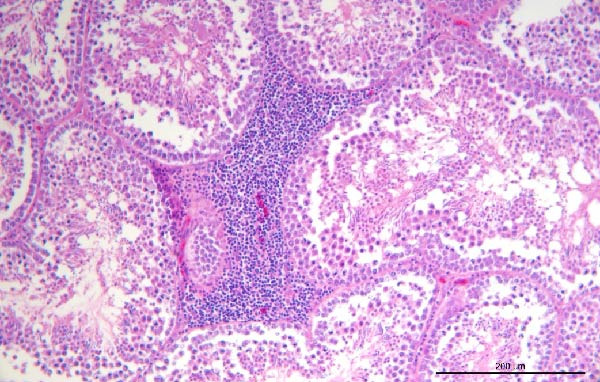
(B)

**Figure:**
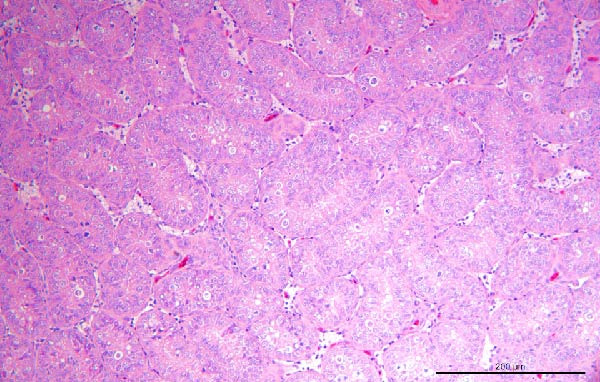
(C)

**Figure:**
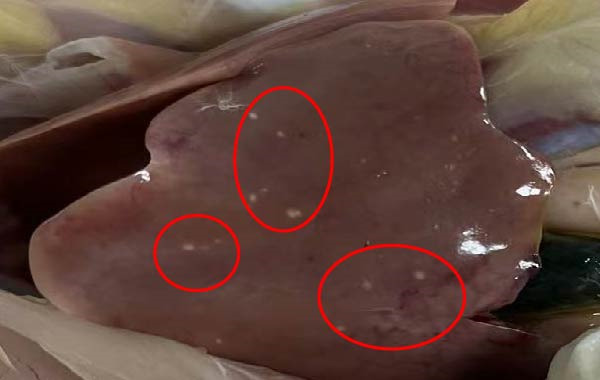
(D)

**Figure:**
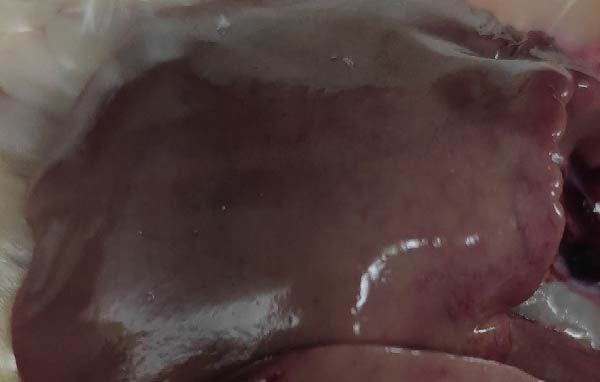
(E)

**Figure:**
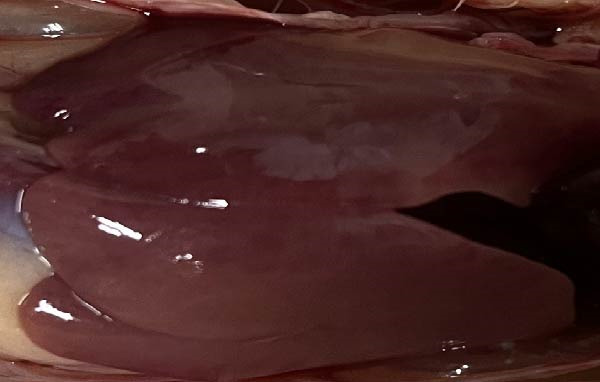
(F)

**Figure:**
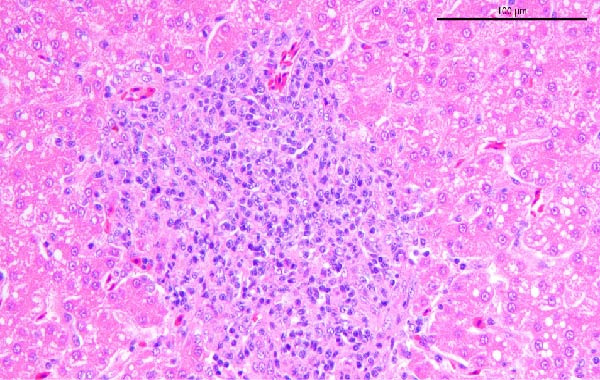
(G)

**Figure:**
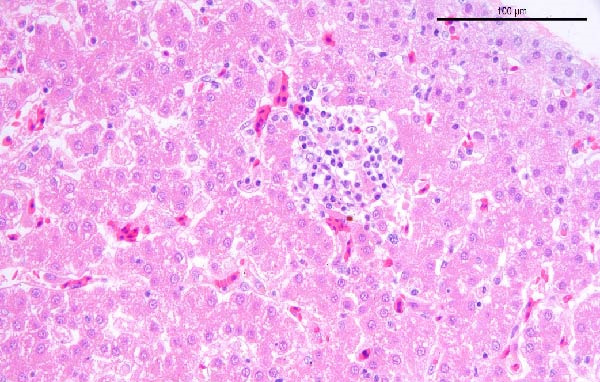
(H)

**Figure:**
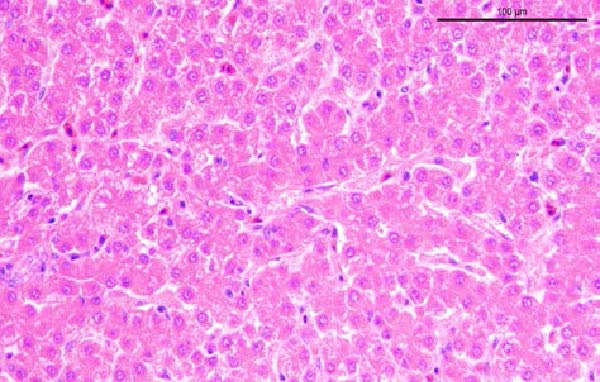
(I)

Figure 5The results of the animal experiments are shown with a dot plot. (A–D) Body weights and ratios of immune organ weight to body weight in each group at 7, 14, 21, 28, and 90 dpc. Data are presented as mean ± SD (*n* = 3 per group at 7–28 dpc; *n* = 6 per group at 90 dpc). Statistical significance was defined as *p* < 0.05 ( ^∗^). (E–H) Normalized viral loads in immune organs and feather pulp of chickens from the different groups at 7, 14, 21, 28, and 90 dpc. Normalized viral loads were calculated as the logarithmic values of Marek’s disease virus copy numbers per million cells. Statistical significance was defined as follows:  ^∗^ *p* < 0.05,  ^∗∗^ *p* < 0.01,  ^∗∗∗^ *p* < 0.001, and  ^∗∗∗∗^ *p* < 0.0001.

**Figure:**
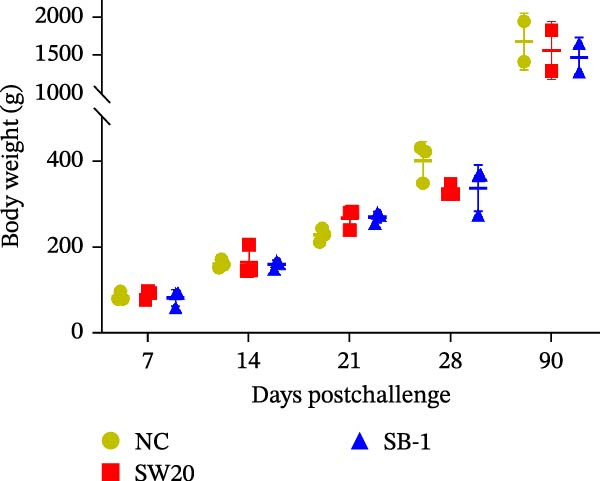
(A)

**Figure:**
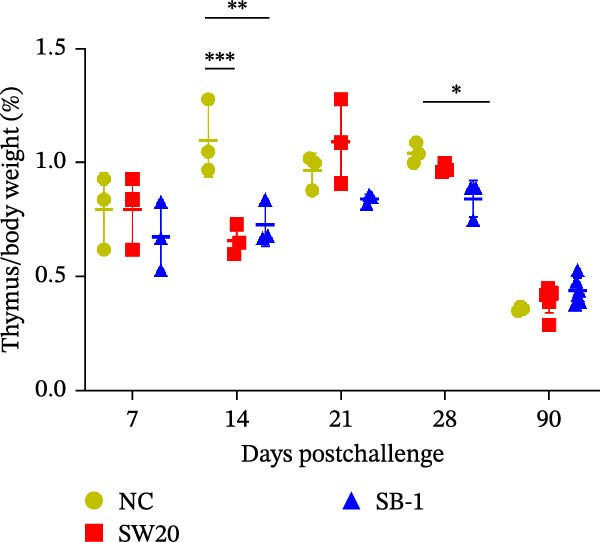
(B)

**Figure:**
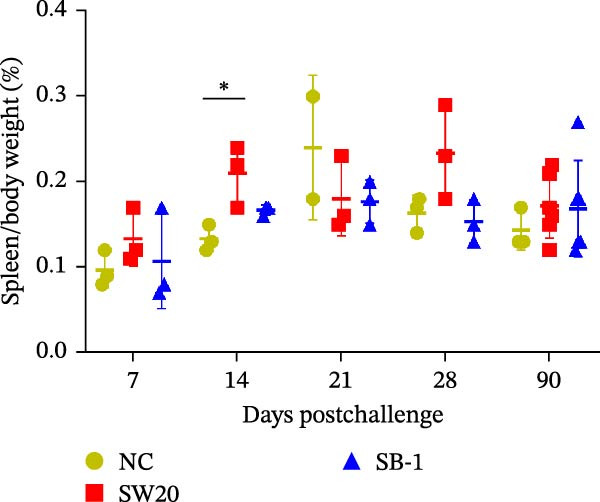
(C)

**Figure:**
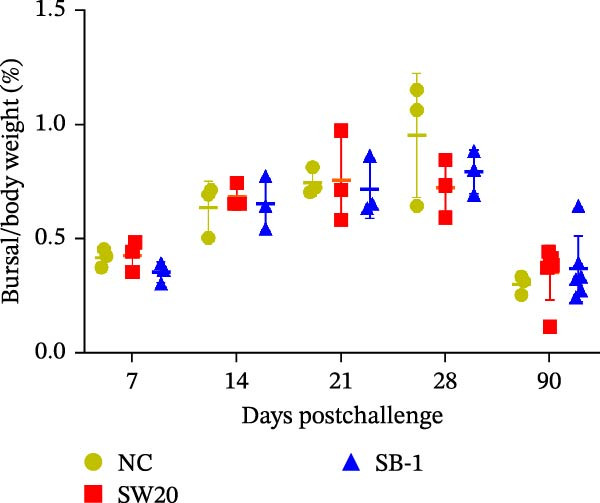
(D)

**Figure:**
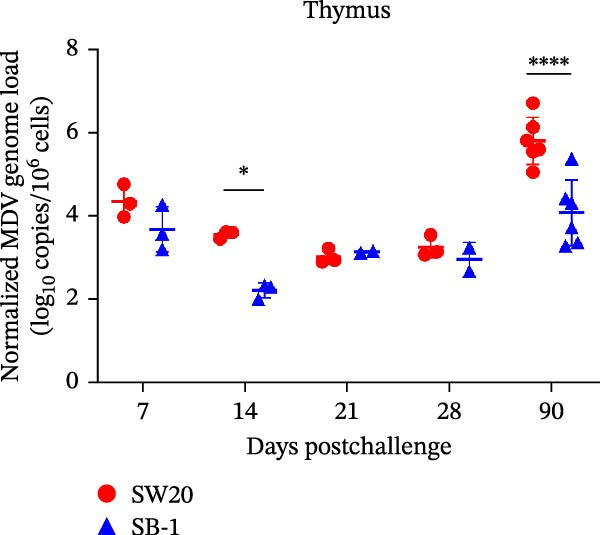
(E)

**Figure:**
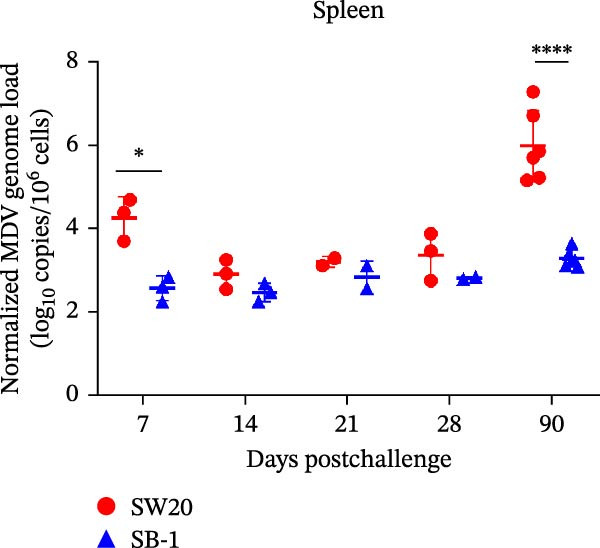
(F)

**Figure:**
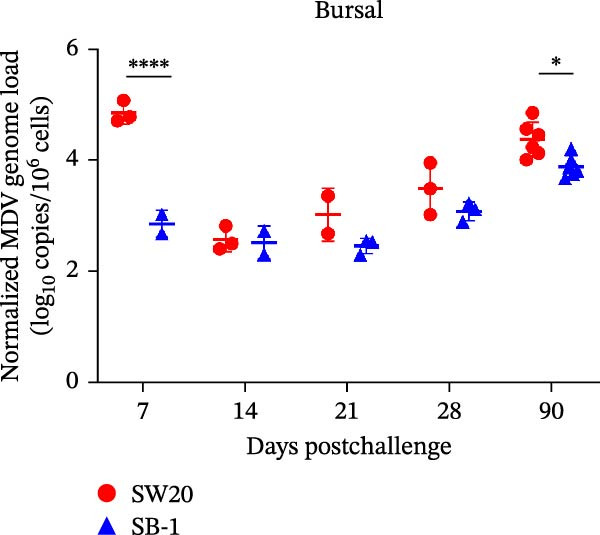
(G)

**Figure:**
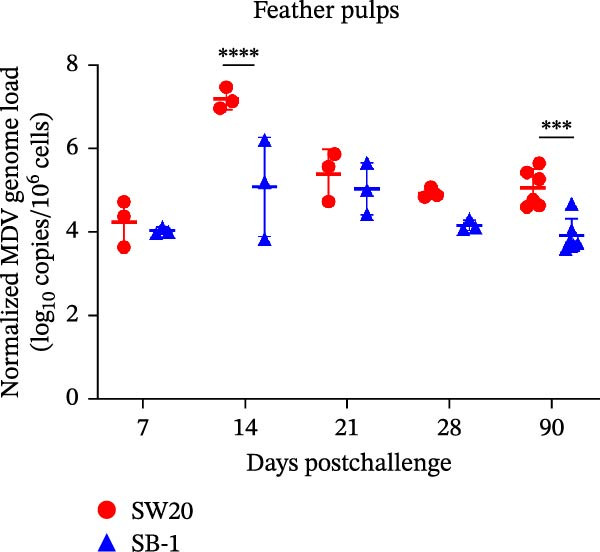
(H)

To compare the replication ability of MDV‐2 in vivo, viral loads in immune organs (thymus, spleen, and bursa) and feather pulp were detected and analyzed at five different time points (Figure 5E–H). A marked difference was observed, with viral load in the spleen and bursa of SW20‐infected chickens being significantly higher than that in the same organs of SB‐1‐infected chickens at 7 dpc (*p*  < 0.05). Similar results were noted in the thymus and feather pulp at 14 dpc (*p*  < 0.05). In addition, at 90 dpc, replication titers in the thymus, spleen, bursa, and feather pulp were significantly higher in SW20‐infected chickens than in SB‐1‐infected chickens (*p*  < 0.05).

Taken together, these findings indicate that SW20 replicates more efficiently in vivo than SB‐1, as evidenced by higher viral loads, and is associated with thymic atrophy, spleen enlargement, and lymphocytic hepatitis in chickens.

## 4. Discussion

Despite the use of MDV vaccines, MD continues to occur, underscoring the need for ongoing surveillance of the disease. In this study, 150 MD‐vaccinated chicken flocks across 21 provinces and four municipalities in China were tested for MDV‐1. At the same time, all flocks tested positive for MDV‐2 by PCR, with the proportion of positive flocks ranging from 20% to 100% between 2015 and 2024. However, the role of MDV‐2 in coinfections in chickens remains unclear, necessitating its isolation and further characterization.

In this study, strains were isolated from positive flocks, identified, and analyzed. To date, no genome of a Chinese MDV‐2 strain has been published. Phylogenetic analysis revealed that four strains (SW20, JLWK1501, LB3, and ZH4) belong to MDV‐2 and share greater homology with the US strain 301B/1 than with other MDV‐2 reference strains. Compared with foreign MDV‐2 reference strains, 33 genes of the Chinese strain exhibited length differences, and 27 genes displayed SNPs when compared with 301B/1. These findings indicate that the MDV‐2 strains vary across countries. Notably, four genes (*ORF3*, *ORF413*, *R-LORF1*, *and R-LORF9*) in the Chinese MDV‐2 strains differed from those in the 301B/1 strain, with distinct length variations. Among these genes, *ORF413* encodes a virion coat protein, whereas the functions of the other proteins remain unknown. Additionally, proteins encoded by these genes may influence virus replication due to SNPs, including gH (*UL22*), gL (*US7*), large tegument protein (*UL36*), multiply hydrophobic protein (*UL43*), tegument protein (*UL47*), hypothetical protein (*ORF473*), DNA helicase/primase complex (*UL52*), major IE transactivator (*ICP4*), protein kinase (*US3*), DNA polymerase (*UL56*), and virion protein (*US10*) [47], along with unknown‐function proteins LORF4, R‐LORF4, UL17, UL16, and UL33. Our data provide strong evidence that the hypervariable region in the Chinese strain is primarily located in the repetitive region and that these gene changes may affect virus replication, consistent with the observed characteristics of Chinese MDV‐2 strains. Although the SB‐1 bivalent vaccine has been used in China [48], the currently isolated strains show significant genetic divergence from SB‐1 and lack an LTR. In contrast, the 301B/1 strain has not been used in China. These findings suggest that the prevailing field strains did not originate directly from the vaccine strains. However, whether these strains evolved from vaccine strains remains uncertain.

Despite this, little is known about the role of MDV‐2 in poultry flocks. In this study, edema and congestion in the liver of one chicken infected with SW20 (China) were observed, accompanied by gray–white necrotic foci. Combined with the pathological findings, these lesions were identified as inflammatory nodules. This experiment showed that no chickens died by 90 dpc, indicating that the Chinese MDV‐2 strain induces inflammatory nodules but does not cause mortality or growth retardation. Previous studies have shown that MDV‐1 can cause a 200%–700% increase in the size of testes [49, 50, 51]. The SB‐1 strain of MDV‐2 has been reported to increase the incidence of lymphoid leukemia induced by exogenous ALV [52], induce spontaneous avian leukemia virus‐like lymphoma in ALVA6 transgenic chickens [53], and significantly elevate the incidence of REV. These findings indicate a potential threat posed by coinfection with other viruses in the poultry industry. To date, no pathogenic or tumorigenic cases of MDV‐2 have been documented. Existing commercial MD vaccines are designed exclusively for the prevention of MDV‐1. The thymic atrophy, spleen enlargement, and lymphocytic hepatitis caused by the prevailing MDV‐2 strains in China may pose a significant threat to the poultry industry, potentially leading to significant economic losses through prolonged feeding periods and increased reliance on vaccines and therapeutic interventions.

Both SW20 and SB‐1 also induced thymus atrophy between 7 and 21 dpc [54] resulting from cytolytic damage [55]. However, the degree of thymic damage was more severe in SW20‐infected chickens than in SB‐1‐infected chickens (*p*  < 0.05), a pattern that aligns with the higher viral load detected in immune organs. SW20 also induced splenic enlargement at 14 dpc, which is consistent with the known biological response to MDV‐1 infection [56]. Furthermore, the viral load in feather pulp proved ideal for noninvasive sampling to detect and measure infection in chickens. Our results showed that SW20 demonstrated higher replication capacity during the cytolytic infection phase, with replication gradually decreasing during the latent infection period. However, by 28 dpc, the replication titer increased, indicating a transition to later infection stages. In contrast, SB‐1 showed a sustained decline in replication levels beginning at 14 dpc. These findings indicate that SW20 displays detectable virulence in infected chickens, providing further insight into the pathogenic characteristics of MDV‐2. Whether MDV‐2 can induce tumorigenesis, as well as the mechanisms underlying potential tumorigenic outcomes, remains to be elucidated.

## 5. Conclusions

The epidemiology of MDV‐2 was systematically investigated in this study, indicating that MDV‐2 shows an expanding dissemination trend in China and is prevailing in commercial flocks. MDV‐2 strains can cause thymus atrophy, spleen enlargement, and testicular swelling. Therefore, continued epidemiological surveillance and reinforced biosecurity measures against MDV‐2 remain critical for preventing and controlling the disease.

## Funding

This study was supported by the National Natural Science Foundation of China (Grants 32170170 and U21A20260) and earmarked funds from the China Agriculture Research System (Grant CARS‐41).

## Conflicts of Interest

The authors declare no conflicts of interest.

## Supporting Information

Additional supporting information can be found online in the Supporting Information section.

## Supporting information


**Supporting Information** Table S1. Sequence of TaqMan probes and primers used in the present study. Table S2. Detailed information for all MDV‐2 isolates obtained in this study, including flock origin, year of detection, coinfection status, and CPE characteristics. Table S3. List of SNPs identified in the 27 genes showing sequence variation relative to the 301B/1, SB‐1, and HPRS24 reference genomes.

## Data Availability

The nucleotide sequences generated in this study were submitted to GenBank (NCBI) under the following Accession Numbers: PX425604 (SW20), PX425602 (JLWK1501), PX425603 (LB3), and PX425605 (ZH4).
