# Supplementary material for: Epidemiology of Marek’s Disease Virus Type 2 and the Molecular and Pathology Characterization of Virus Isolates in China (2015–2024)
Source: Transbound Emerg Dis. 2026 Apr 10;2026:5910446. doi: 10.1155/tbed/5910446 (PMC13066712; doi:10.1155/tbed/5910446)
Supplement: Supplementary file 1 — Supporting Information Table S1. Sequence of TaqMan probes and primers used in the present study. Table S2. Detailed information for all MDV‐2 isolates obtained in this study, including flock origin, year of detection, coinfection status, and CPE characteristics. Table S3. List of SNPs identified in the 27 genes showing sequence variation relative to the 301B/1, SB‐1, and HPRS24 reference genomes. [file TBED-2026-5910446-s001.docx]

Supplementary Table

Table 1 Sequence of TaqMan probes and primers used to in the present study

| number | Gene | Primer name | Primer sequence (5'-3') |
| --- | --- | --- | --- |
| 1 | Meq | Pmeq-F | GGGAAATGACAGGTGAATTGTG |
|  |  | Pmeq-R | TAAGGAAAATTTGTTACCCCAG |
| 2 | ORF873 | ORF873-F | TACAAATTCAATCTCCGGGTT |
|  |  | ORF873-R | GTGTCTATAGGTTCCGCACCA |
| 3 | US3 | MDV-3-F | ATGGAAGTAGATGTTGAGTCTTCG |
|  |  | MDV-3-R | CGATATACACGCATTGCCATACAC |
| 4 | VP3 | CIAV F | AATGAACGCTCTCCAAGAAG |
|  |  | CIAV R | AGCGGATAGTCATAGTAGAT |
| 5 | env | ALV-F | CGGAGAAGACACCCTTGCT |
|  |  | ALV-AR | GCATTGCCACAGCGGTACTG |
|  |  | ALV-BR | GTAGACACCAGCCGGACTATC |
|  |  | ALV-JR | CGAACCAAAGGTAACACACG |
| 6 | LTR | REV F | GCCTTAGCCGCCATTGTA |
|  |  | REV R | CCAGCCAACACCACGAACA |
| 7 | Hexon | FAdV F | GCCACCGGAAGCTACTTTGA |
|  |  | FAdv R | TTGTGATCCATGGGCATGA |
| 8 | ovo | ovo F | CACTGCCACTGGGCTCTGT |
|  |  | ovo R | GCAATGGCAATAAACCTCCAA |
|  |  | Probe | 5'-(ROX) AGTCTGGAGAAGTCTGTGCAGCCTCCA (BHQ-2)-3’ |
| 9 | DNA-Pol (MDV2) | MDV-2 F | AGCATGCGGGAAGAAAAGAG |
|  |  | MDV-2 R | GAAAGGTTTTCCGCTCCCATA |
|  |  | Probe | 5’-(ROX) CGCCCGTAATGCACCCGTGACT (BHQ-2)-3’ |

Table 2 Isolation of virus in this study

| Number | Name | Year | Chicken | Serotype | Place |
| --- | --- | --- | --- | --- | --- |
| 1 | SW1 | 2021 | Layer | MDV-2 | Fujian |
| 2 | SW2 | 2021 | Layer | MDV-2 | Fujian |
| 3 | SW3 | 2021 | Layer | MDV-2 | Fujian |
| 4 | SW4 | 2021 | Layer | MDV-2 | Fujian |
| 5 | SW5 | 2021 | Layer | MDV-2+HVT | Fujian |
| 6 | SW6 | 2021 | Layer | MDV-2 | Fujian |
| 7 | SW7 | 2021 | Layer | MDV-2 | Fujian |
| 8 | SW8 | 2021 | Layer | MDV-2 | Fujian |
| 9 | SW9 | 2021 | Layer | MDV-2+HVT | Fujian |
| 10 | SW10 | 2021 | Layer | MDV-2 | Fujian |
| 11 | SW11 | 2021 | Layer | MDV-2+HVT | Fujian |
| 12 | SW12 | 2021 | Layer | MDV-2 | Fujian |
| 13 | SW13 | 2021 | Layer | MDV-2+HVT | Fujian |
| 14 | SW14 | 2021 | Layer | MDV-2 | Fujian |
| 15 | SW15 | 2021 | Layer | MDV-2 | Fujian |
| 16 | SW16 | 2021 | Layer | MDV-2 | Fujian |
| 17 | SW17 | 2021 | Layer | MDV-2+HVT | Fujian |
| 18 | SW18 | 2021 | Layer | MDV-2+HVT | Fujian |
| 19 | SW19 | 2021 | Layer | MDV-2+HVT | Fujian |
| 20 | SW20 | 2021 | Layer | MDV-2 | Fujian |
| 21 | SW21 | 2021 | Layer | MDV-2+HVT | Fujian |
| 22 | SW24 | 2021 | Layer | MDV-1+MDV-2 | Fujian |
| 23 | JLWK1501 | 2022 | Layer | MDV-2 | Jilin |
| 24 | JLWK1502 | 2022 | Layer | MDV-1+MDV-2 | Jilin |
| 25 | JLWK1503 | 2022 | Layer | MDV-2 | Jilin |
| 26 | JLWK1504 | 2022 | Layer | MDV-2 | Jilin |
| 27 | JLWK1505 | 2022 | Layer | MDV-2 | Jilin |
| 29 | JLWK1403 | 2022 | Layer | MDV-2 | Jilin |
| 30 | DKH105 | 2022 | Layer | MDV-2 | Fujian |
| 31 | DKH106 | 2022 | Layer | MDV-2 | Fujian |
| 32 | DKH5019 | 2022 | Layer | MDV-2 | Fujian |
| 33 | DKH5025 | 2022 | Layer | MDV-2 | Fujian |
| 34 | WYH1039 | 2022 | Layer | MDV-2 | Fujian |
| 35 | FJSN02 | 2022 | Layer | MDV-2 | Fujian |
| 36 | FJSN05 | 2022 | Layer | MDV-2 | Fujian |
| 37 | FJSN06 | 2022 | Layer | MDV-2 | Fujian |
| 38 | FJSN07 | 2022 | Layer | MDV-2 | Fujian |
| 39 | FJSN09 | 2022 | Layer | MDV-2 | Fujian |
| 40 | FJSN11 | 2022 | Layer | MDV-2 | Fujian |
| 41 | FJSN12 | 2022 | Layer | MDV-2 | Fujian |
| 42 | FJSN17 | 2022 | Layer | MDV-2 | Fujian |
| 43 | FJSN19 | 2022 | Layer | MDV-2 | Fujian |
| 44 | FJSN21 | 2022 | Layer | MDV-1+MDV-2 | Fujian |
| 45 | FJSN25 | 2022 | Layer | MDV-2 | Fujian |
| 46 | FJSN26 | 2022 | Layer | MDV-2 | Fujian |
| 47 | FJSN31 | 2022 | Layer | MDV-2 | Fujian |
| 48 | FJSN32 | 2022 | Layer | MDV-2 | Fujian |
| 49 | FJSN34 | 2022 | Layer | MDV-2 | Fujian |
| 50 | FJSN39 | 2022 | Layer | MDV-2 | Fujian |
| 51 | FJSN43 | 2022 | Layer | MDV-2 | Fujian |
| 52 | FJSN44 | 2022 | Layer | MDV-2 | Fujian |
| 53 | LB1 | 2023 | Layer | MDV-2+HVT | Heilongjiang |
| 54 | LB3 | 2023 | Layer | MDV-2 | Heilongjiang |
| 55 | LH19 | 2023 | Layer | MDV-2 | Heilongjiang |
| 56 | ZH4 | 2023 | Layer | MDV-2 | Heilongjiang |
| 57 | ZH5 | 2023 | Layer | MDV-2 | Heilongjiang |
| 58 | NM401 | 2023 | Layer | MDV-2 | Inner Mongolia |
| 59 | NM403 | 2023 | Layer | MDV-2 | Inner Mongolia |
| 60 | NM405 | 2023 | Layer | MDV-2 | Inner Mongolia |
| 61 | NM502 | 2023 | Layer | MDV-2 | Inner Mongolia |
| 62 | NM509 | 2023 | Layer | MDV-2 | Inner Mongolia |
| 63 | NM603 | 2023 | Layer | MDV-2 | Inner Mongolia |
| 64 | NM702 | 2023 | Layer | MDV-2 | Inner Mongolia |
| 65 | NM1004 | 2023 | Layer | MDV-2 | Inner Mongolia |
| 66 | NM1103 | 2023 | Layer | MDV-2 | Inner Mongolia |

Table3 The SNPs of twenty-seven genes among the Chinese MDV-2 genomes with the corresponding genes in the strains of 301B/1, SB-1 and HPRS24 genome.

|  | Amino acid sequence | | | | |  |  | Position in Amino acid |
| --- | --- | --- | --- | --- | --- | --- | --- | --- |
| ORFs | SW20 | JLWK1501 | Hrb09LB3 | Hrb09ZH4 | 301B/1 | SB-1 | HPRS24 |  |
| ICP4 | P | P | L | L | L | P | P | 252 |
| ICP4 | H | L | H | H | H | H | H | 260 |
| ICP4 | T | S | T | T | T | T | T | 261 |
| ICP4 | N | N | N | N | N | N | D | 417 |
| ICP4 | Q | Q | Q | Q | Q | Q | R | 1133 |
| ICP4 | V | V | V | V | V | V | A | 1379 |
| ICP4 | Q | Q | Q | Q | Q | Q | R | 1782 |
| ICP4 | R | R | C | C | C | R | R | 1982 |
| LORF1 | E | E | E | E | E | E | D | 345 |
| LORF1 | K | K | K | K | K | K | E | 371 |
| R-LORF4 | V | G | V | V | V | V | V | 39 |
| R-LORF4 | V | V | L | L | L | L | L | 75 |
| R-LORF4 | L | L | L | L | L | L | P | 128 |
| UL1 | R | R | H | H | H | H | H | 2 |
| UL8 | R | Q | R | R | R | R | R | 555 |
| UL16 | M | V | V | V | V | V | V | 349 |
| UL17 | K | R | R | R | R | R | R | 450 |
| UL19 | I | I | I | I | I | I | T | 597 |
| UL19 | D | D | D | D | D | D | G | 754 |
| UL19 | R | R | R | R | R | R | K | 884 |
| UL22 | S | S | N | N | N | N | N | 762 |
| UL26 | L | L | L | L | L | L | W | 238 |
| UL29 | I | I | I | I | I | I | M | 189 |
| UL32 | A | A | A | A | A | A | C | 533 |
| UL33 | A | T | A | A | A | A | A | 3 |
| UL33 | T | A | T | T | T | T | T | 90 |
| UL38 | A | A | A | A | A | A | R | 87 |
| UL38 | A | A | A | A | A | A | H | 147 |
| UL40 | A | A | A | A | A | A | R | 214 |
| UL41 | R | R | R | R | R | R | P | 138 |
| UL42 | H | H | H | H | H | H | Q | 299 |
| UL43 | A | T | A | A | A | A | A | 184 |
| UL43 | A | A | A | A | A | A | L | 212 |
| UL43 | I | I | I | I | I | I | F | 213 |
| UL43 | H | H | H | H | H | H | T | 214 |
| UL43 | V | V | V | V | V | V | W | 215 |
| UL43 | V | V | V | V | V | V | S | 216 |
| UL43 | R | R | R | R | R | R | E | 217 |
| UL43 | D | D | D | D | D | D | T | 218 |
| UL43 | A | A | A | A | A | A | P | 219 |
| UL43 | S | S | S | S | S | S | V | 220 |
| UL43 | R | R | R | R | R | R | G | 221 |
| UL44 | K | K | K | K | K | K | P | 247 |
| UL45 | R | R | R | R | R | R | P | 10 |
| UL45 | D | D | D | D | D | D | R | 11 |
| UL45 | V | V | V | V | V | V | G | 13 |
| UL45 | A | A | A | A | A | A | P | 14 |
| UL48 | G | G | G | G | G | R | G | 224 |
| UL48 | H | H | H | H | H | H | D | 276 |
| UL51 | Q | Q | Q | Q | Q | Q | H | 106 |
| UL51 | R | R | R | R | R | R | E | 107 |
| UL51 | M | M | M | M | M | M | V | 108 |
| UL52 | T | A | A | A | A | A | A | 139 |
| UL52 | K | K | R | R | R | K | K | 349 |
| UL52 | I | M | I | I | I | I | I | 753 |
| UL53 | A | A | A | A | A | A | G | 230 |
| US3 | P | P | P | L | P | P | P | 31 |
| US3 | P | P | P | P | P | P | R | 165 |
| US7 | I | I | M | M | M | M | M | 346 |
| US10 | Y | Y | C | C | C | Y | Y | 203 |
